# Prioritizing nurturing care at the municipal and district level with the Brazilian Early Childhood Friendly Municipal Index (IMAPI)

**DOI:** 10.1111/mcn.13312

**Published:** 2022-03-07

**Authors:** Gabriela Buccini, Juliana L. Pimentel, Jéssica Pedroso, Stefanie Eugênia dos Anjos Coelho Kubo, Juracy Bertoldo, Alberto Sironi, Marcos E. Barreto, Rafael Pérez‐Escamilla, Muriel B. Gubert

**Affiliations:** ^1^ Department of Social and Behavioral Health, School of Public Health University of Nevada Las Vegas Nevada USA; ^2^ Departamento de Nutrição Universidade de Brasilia Brasília Brazil; ^3^ Departamento de Ciência da Computação Universidade Federal da Bahia Salvador Brazil; ^4^ Department of Social and Behavioral Sciences Yale School of Public Health New Haven Connecticut USA

**Keywords:** Brazil, child development, cities, monitoring, nurturing care, prioritization

## Abstract

The Brazilian Early Childhood Friendly Municipal Index (IMAPI) is a population‐based approach to monitor the nurturing care environment for early childhood development (ECD) using routine information system data. It is unknown whether IMAPI can be applied to document metropolitan urban territorial differences in nurturing care environments. We used Brasilia, Brazil's capital with a large metropolitan population of 2,881,854 inhabitants divided into 31 districts, as a case study to examine whether disaggregation of nurturing care data can inform a more equitable prioritization for ECD in metropolitan areas. IMAPI scores were estimated at the municipal level (IMAPI‐M, 31 indicators) and at the district level (IMAPI‐D, 29 indicators). We developed a quantitative prioritization process for indicators in each IMAPI analysis, and those selected were jointly mapped in the socioecological model for the role of indicators in relation to the enabling environment for nurturing care. Out of 28 common nurturing care indicators across IMAPI analysis, only four were prioritized in both analyses: one from the Adequate nutrition, two from the Opportunities for early learning, and one from the Responsive caregiving domains. These four indicators were mapped as enabling policies, supportive services, and caregivers’ capabilities (socioecological model) and Effort, Coverage, and Quality (indicator's role). In conclusion, the different levels of nurturing care data disaggregation in the IMAPI can better inform decision‐making than each one individually, especially in metropolitan areas where municipalities and districts within metropolitan areas have relative decision‐making autonomy.

## Key messages

- The Brazilian Early Childhood Friendly Municipal Index (IMAPI) is an innovative population‐based approach created to systematically measure nurturing care for early childhood development (ECD) using routine information systems data at the municipal level.
- Brasilia was used as a case study to evaluate whether different levels of nurturing care data disaggregation (municipal vs. district level) can inform more equitable prioritization for ECD investments in metropolitan areas.
- We developed a quantitative prioritization process of indicators to analyse IMAPI at the municipal and district levels, and the selected indicators were jointly mapped into the socioecological model, specifying the roles of the indicator in the enabling environment for nurturing care.
- The two different levels of data disaggregation of the IMAPI can better inform nurturing care‐related decisions than each individual level in metropolitan areas where municipalities and districts within metropolitan areas have relative decision‐making autonomy.

## Graphical abstract

The Brazilian Early Childhood Friendly Municipal Index (IMAPI) is an innovative population‐based approach created to systematically measure nurturing care for early childhood development (ECD) using routine information systems data at the municipal level. Brasilia was used as a case study to evaluate whether different levels of nurturing care data disaggregation (municipal vs. district level) can inform more equitable prioritization for ECD investments in metropolitan areas. The two different levels of data disaggregation of the IMAPI can better inform nurturing care‐related decisions than each individual level in metropolitan areas where municipalities and districts within metropolitan areas have relative decision‐making autonomy.

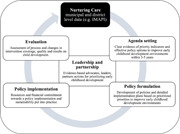

## 1 BACKGROUND

The Nurturing Care Framework (NCF) outlines five comprehensive and interrelated evidence‐based components (i.e., health, nutrition, early learning, security and safety, and responsive caregiving) that are essential for proper child growth and development and necessary for countries to attain the Sustainable Development Goals (SDGs) (Black et al., 2017, 2021; Britto et al., 2017). Enabling nurturing care environments with evidence‐based policies, programmes, and services to transform the health and human potential of current and next generations require focused evidence‐informed investments in policies and programmes accompanied by sound governance and monitoring systems. Monitoring the implementation of the NCF using data collected from population‐level surveys, censuses, or administrative databases is now considered a global priority (Operationalizing Nurturing Care for Early Childhood Development, 2020).

**Figure 1:**
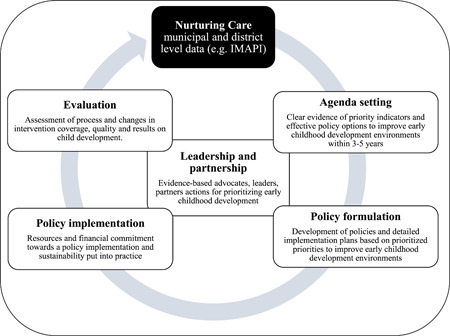
Heuristic Policy Process used to frame how municipal and district‐level data on nurturing care can accelerate inform key processes in early childhood development policies

In response, several population‐level approaches based on a set of nurturing care related‐indicators have been developed to monitor the nurturing care environment at different levels of governance, that is, country, state, and municipal levels (Pedroso et al., 2021). Nevertheless, a significant gap remains on whether the nurturing care‐related data compiled by these population‐level approaches are useful to inform ECD policy processes (Shawar & Shiffman, 2017). Based on the heuristic policy model, our theory of change hypothesizes that using nurturing care‐related data can support ECD policy processes by generating evidence on priority nurturing care indicators, which in turn facilitates policy formulation and adoption, increases Coverage and Quality of interventions and ultimately results in enabling nurturing care environments for ECD (Figure 1). Leadership and partnership support all aspects of the heuristic nonlinear policy process (Black et al., 2017; Darmstadt et al., 2014). Frameworks and participatory methods have previously been used to prioritize research (Dechartres & Ravaud, 2015; MacFarlane et al., 2017; Minelli & Baio, 2015) and to set a global research agenda for ECD (Sharma et al., 2017). Evidence on principles that influence policy prioritization in developing countries may include developing shared policy goals, identifying smart strategies, assessing policy compatibility, aligning policy instruments, and factoring sustainability into short‐ and long‐term policy decisions (Rasul, 2020). However, a standard methodology is still unavailable that could be employed to improve the ECD policy process by using nurturing care‐related data to prioritize investments that ultimately enable nurturing care environments.

In Brazil, the largest country in Latin America and the Caribbean, and other Latin American countries with similar governance structures, municipalities must develop their own ECD implementation plans following local policy decision‐making roadmaps to prioritize investments to fight nurturing care inequities (Avellaneda, 2013). Therefore, this highlights the need to develop the Brazilian Early Childhood Friendly Municipal Index (IMAPI—*Índice de Município Amigo da Primeira Infância*), which is a validated population‐based approach to capture the strength of the multiple dimensions of nurturing care for ECD using data from routine information systems at the municipal level (Buccini, Kubo, et al., 2021; Buccini, Pedroso, et al., 2021). Construct validity (Buccini, Pedroso, et al., 2021) as well as predictivity and concurrent validity (Buccini, Kubo, et al., 2021) of IMAPI has confirmed its potential to detect inequities in nurturing care at the municipal level (Buccini, Kubo, et al., 2021). On the other hand, it is unknown whether IMAPI can be applied to document territorial differences of a large metropolitan urban in nurturing care environments where a high degree of maternal‐child health and nutrition inequities exist (Matthews et al., 2010; Vilar‐Compte et al., 2021).

We focused our work on Brasilia which is Brazil's capital and has a large metropolitan population of 2,881,854 inhabitants living across 31 districts. This focus allows us to test whether disaggregation of nurturing care data at the municipal and district level could inform sound decision‐making, prioritization, and accountability process by ECD policymakers. Our objective was twofold: (i) to develop a quantitative prioritization process of nurturing care indicators to analyse IMAPI at municipal and district level and (ii) to examine divergence and convergence of two levels of data disaggregation to inform more equitable prioritization for decision‐making about ECD in a Brazilian metropolitan area.

## 2 METHODS

### 2.1 Study setting

**Figure 2:**
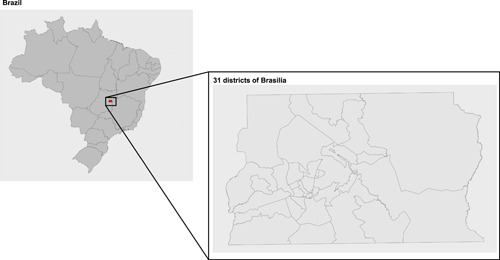
The geographic location of Brasilia and its 31 districts

Brasilia (also known as the Federal District) is the capital of Brazil. This municipality located in the Central‐Western region of the country has a large population of 2,881,854 inhabitants divided into 31 districts (Figure 2). In 2019, 57.6% of the population identifying as Black or Brown race‐skin colour, and 11.2% of the population lived in moderate or extreme poverty. Since 2018, Brasilia has conducted a standardized household population‐level survey collected every 2 years that offers information disaggregated at the district level (*PDAD*—*Pesquisa Distrital Por Amostra de Domicílios*—*CODEPLAN*, 2018). Therefore, Brasilia can provide an important case study to understand whether having nurturing care indicators at the municipal versus district level improves equitable decision‐making for ECD.

### 2.2 Data source

The overall scores and nurturing care domains subscores of IMAPI were calculated for both the municipality of Brasilia and the 30 districts within the municipality using the IMAPI eight‐step methodology described in detail elsewhere (Buccini, Kubo, et al., 2021; Buccini, Pedroso, et al., 2021). For these analyses, data for the municipality of Brasilia were extracted from IMAPI computed for all Brazilian municipalities (IMAPI‐M) and data for districts were extracted from IMAPI computed for districts within Brasilia (IMAPI‐D). In both cases, SMART analytical weights were attributed to each indicator to improve reliability when generating IMAPI indexes (i.e., higher analytical weight was attributed to indicators with better data quality). Following the statistical criteria of having at least two indicators in the subscore domain to be included in the overall IMAPI score, the responsive caregiving domain was excluded. The overall IMAPI score is composed of four nurturing care domains. The overall IMAPI score and subscores of the nurturing care domains ranged from 0 to 100, and the scores were categorized in high, medium, and low categories based on the corresponding tercile distributions. Methodological differences between IMAPI‐M (municipal level) and IMAPI‐D (district level) analysis are described below.

#### 2.2.1 IMAPI at municipal level (IMAPI‐M)

The overall IMAPI‐M score is composed of 30 indicators across four Nurturing Care domains: Good health (n = 14), Adequate nutrition (4), Opportunities for early learning (7), and Security and Safety (5). Indicator names and definitions can be found in Supporting Information Appendix 1. We extracted the information of the overall IMAPI score and domains subscores, and the scores of nurturing care indicators for Brasilia municipality through the IMAPI website (www.imapi.org/en), which provides freely available data.

#### 2.2.2 IMAPI at the district level (IMAPI‐D)

To make IMAPI‐D comparable to the IMAPI‐M, we used the list of 30 nurturing care indicators as a basis to select the same indicators at the district level. In the process of collecting data, two indicators, not included in the IMAPI‐M due to the lack of data, were included in the Security and Safety domain of the IMAPI‐D: (1) Water system supply (i.e., percentage of the population with adequate water supply) and (2) Sewage system (i.e., percentage of the population with adequate sewage system). Additionally, three indicators were excluded due to the lack of data at the district level; two in the adequate nutrition domain (severe food insecurity and Coverage of information on child food consumption) and one in the Security and Safety domain (air pollution) (names and definitions of indicators can be found in Supporting Information Appendix 1). Therefore, the overall IMAPI‐D score was composed of 29 indicators across four Nurturing Care domains: Good health (*n* = 14), Adequate nutrition (2), Opportunities for early learning (7), and Security and Safety (6). We extracted the information of the IMAPI‐D score and domain subscores, and the scores of nurturing care indicators for the 31 districts through the IMAPI‐D website (https://distritofederal.imapi.org/en). For this analysis, one district (SIA district) was excluded because it is a commercial nonresidential area. Therefore, the analytical unit of IMAPI‐D was 30 districts.

### 2.3 Data analysis

#### 2.3.1 Analytical framework

We developed the analytical framework described below to support the interpretation of IMAPI scores at the municipal and district levels.

##### 2.3.1.1 Allocation of nurturing care indicators into the socioecological model

**Figure 3:**
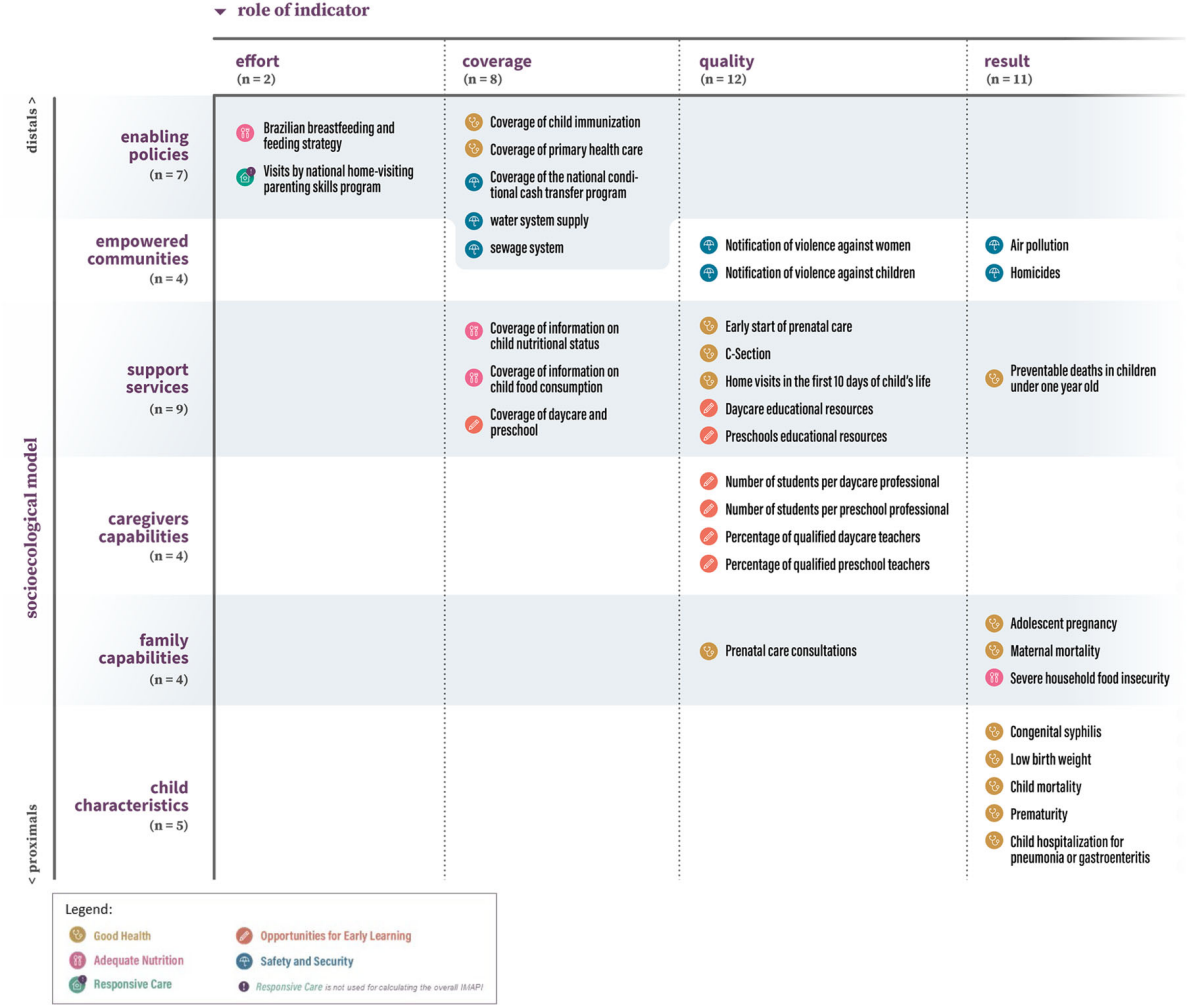
Classification of nurturing care indicators into the socioecological model and the role of the indicator in relation to the enabling environment for nurturing care

We adapted the socioecological model of nurturing care developed by the World Health Organization (Operationalizing Nurturing Care for Early Childhood Development, 2020). Six levels of the socioecological model were operationalized as follows: *Enabling policies* (indicators related to public policies that enable the nurturing care environment); *Empowered com munities* (indicators that characterize the vulnerability of nurturing care within the communities); *Support services* (indicators related to the availability of services and actions that directly impact ECD outcomes); *Caregivers’ capabilities* (indicators related to caregivers’ characteristics and their ability to provide nurturing care); *Family capabilities* (indicators related to families’ characteristics and their ability to provide nurturing care); and *Child characteristics* (indicators related to the biological risk factors to ECD outcomes) (Figure 3, framework). Then, two coauthors with expertise in ECD and cocreators of IMAPI allocated the 31 nurturing care indicators of IMAPI in one of the six levels of the socioecological model (Nurturing Care Framework for Early Childhood Development, 2021a; Operationalizing Nurturing Care for Early Childhood Development, 2020). This allocation was then validated by a senior coauthor who participated in the development of the NCF.

##### 2.3.1.2 Role of the indicators in relation to the enabling environment for nurturing care

Operational definitions of the role of the indicators for enabling ECD environments were developed by the research team after reviewing previous research that conceptualized the performance of health indicators in Brazil and other Latin American countries (Albuquerque & Martins, 2017; Pan American Health Organization, 2018). Each nurturing care indicator was classified into one of four roles: *Effort* (reflects the effort of the municipal management in offering ECD‐related policies, programmes, and services); *Coverage* (reflects the capacity of the ECD system to meet the demand of families for programmes and services); *Quality* (reflects the Quality of ECD‐related programmes and services); and *Result* (reflects the effectiveness of ECD‐related programmes and services) (Figure 3). As with the allocation into the socioecological model, the same two coauthors classified the role of the indicators in relation to the enabling nurturing care environment, then the same senior coauthor validated this allocation.

#### 2.3.2 Quantitative prioritization process

##### 2.3.2.1 Descriptive analysis of IMAPI scores and domain subscores

We conducted a descriptive analysis of nurturing care scores and domain subscores of IMAPI‐M and IMAPI‐D. Districts were characterized by population size—small (up to 20 thousand inhabitants), medium (20–100 thousand inhabitants), and large (more than 100 thousand inhabitants); territory (Central, East, West, North, South); and income groups—low (household income average of R$ 2,472 [~U$ 473]), medium‐low (household income average of R$ 3,101 [~U$ 593]), medium‐high (household income average of R$ 7,266 [~U$ 1,388]), and high (household income average R$ 15,622 [~U$ 2,984]).

##### 2.3.2.2 Prioritization of nurturing care indicators

**Figure 4:**
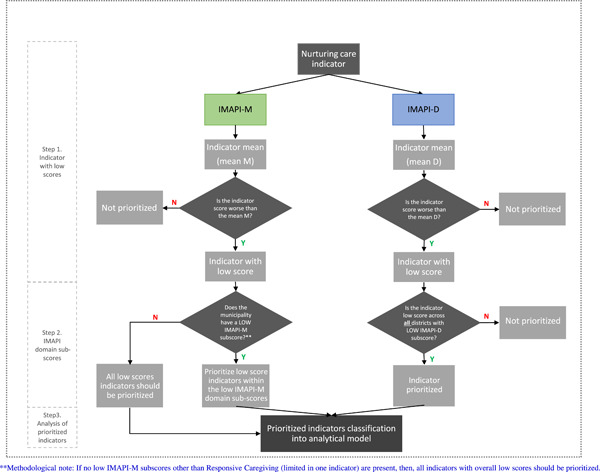
Steps to prioritize nurturing care indicators at the municipal and district levels

The quantitative prioritization process focused on prioritizing nurturing care indicators. Therefore, two important analytical considerations should be noted. First, unweighted indicators were used as each indicator was compared with its mean across municipalities or districts. Second, the single indicator in the responsive caregiving domain was considered in the prioritization process. A decision flowchart guided the data analysis and identification of prioritized nurturing care indicators following three steps: *1*. Indicator with a low score (i.e., identification of indicators with scores worse than the mean); *2*. IMAPI domain subscores (i.e., identification of low score indicators across low domain subscores); and *3*. Analysis of prioritized indicators (i.e., prioritized low scores indicators classified in socioecological model and indicator role) (Figure 4, flowchart). Details of how the decision flowchart was applied to each IMAPI analysis are detailed below.

###### 2.3.2.2.1 IMAPI‐M

In the first step, we computed for each nurturing care indicator the mean across the 5,570 Brazilian municipalities (indicator mean M). Indicators with scores worse than the mean M (indicator with low scores) were then selected. In the second step, we checked for domains with low IMAPI‐M subscores. If there were any, indicators within low IMAPI‐M subscores were prioritized. If no low IMAPI‐M subscores other than Responsive Caregiving (limited to only one indicator) were present, then all indicators with overall low scores were prioritized.

###### 2.3.2.2.2 IMAPI‐D

In the first step, we computed for each nurturing care indicator the mean across the 30 districts (indicator mean D). Indicators with scores worse than the mean D (indicator with low scores) were then selected. In the second step, we identified nurturing care indicators with low scores across all districts with low IMAPI‐D domain subscores (Supporting Information Appendix 2).

###### 2.3.2.2.3 Analysis of prioritized indicators

In the third step, we classified the prioritized indicators in IMAPI‐M and IMAPI‐D into the analytical model. Then, we explored the divergence and convergence of prioritized indicators at both levels of IMAPI and compared the prioritized indicators by nurturing care domain, socioecological model, and role of indicator in relation to the enabling nurturing care environment. Finally, we checked for common indicators prioritized in both levels of IMAPI analyzes.

## 3 RESULTS

### 3.1 Analytical framework

Across the six levels of the socioecological model, most indicators were classified as representing Supportive Services (*n* = 9, 29.0%), followed by Enabling policies and Child characteristics with five (16.1%) indicators in each level, and Empowered communities, Caregivers’ capabilities, and Family capabilities with four (12.9%) indicators in each level (Figure 3, framework). Of the 31 nurturing care indicators of IMAPI‐M, 2 (6.5%) indicators reflect Efforts, 6 (19.3%) Coverage, 12 (38.7%) Quality, and 11 (35.5%) Results in enabling a nurturing care environment for ECD. Water system supply and sewage system, included only in the IMAPI‐D security and safety domain subscore, were both classified as Enabling policies and Coverage.

### 3.2 Quantitative prioritization process

#### 3.2.1 IMAPI‐M

Brasilia had an overall IMAPI‐M score of 55, which we considered to be high in relation to all Brazilian municipalities; it occupied position 184 in the national ranking of 5570 municipalities (Table 1). For the nurturing care domains included in the overall IMAPI‐M, Good health and Security and Safety had medium scores and Adequate nutrition and Opportunities for early learning had high scores in relation to all Brazilian municipalities. Twelve nurturing care indicators with low scores were prioritized: four in the Good health domain (prematurity, low birthweight, congenital syphilis, and Coverage of primary health care), two in the Adequate nutrition domain (Coverage of information on child nutritional status and Coverage of information on child food consumption), three in the Opportunities for early learning (Coverage of daycare and pre‐school, number of students per pre‐school professional, and number of students per daycare professional), two in the Security and Safety domain (notification of violence against women and Coverage of the national conditional cash transfer), and one in the Responsive caregiving (home‐visiting parenting skills programme) (Table 2).

**Table 1:** Descriptive of IMAPI‐M and IMAPI‐D scores and domains subscores in Brasilia municipality and its 30 districts

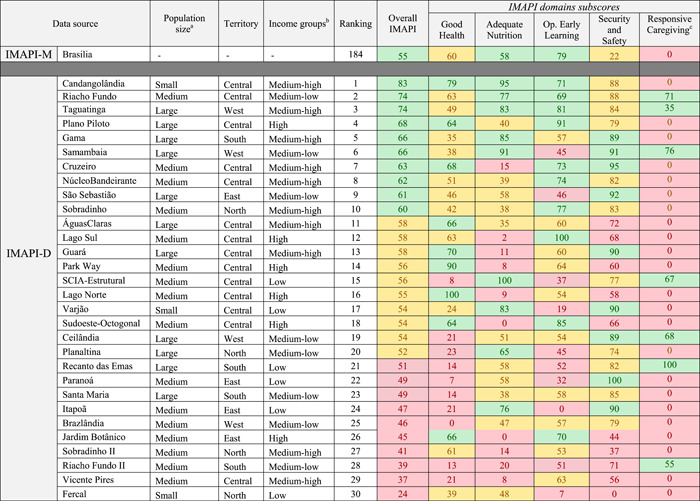

*Note*: Green indicates high IMAPI scores. Yellow indicates medium IMAPI scores. Red indicates low IMAPI scores.

Abbreviation: IMAPI, Brazilian Early Childhood Friendly Municipal Index.

^a^Population size: small (up to 20 thousand inhabitants), medium (20 to 100 thousand inhabitants), large (more than 100 thousand inhabitants).

^b^Income groups: low (household income average of R$ 2,472 [~U$ 473]), medium‐low (household income average of R$ 3,101 [~U$ 593]), medium‐high (household income average of R$ 7,266 [~U$ 1,388]), and high (household income average R$ 15,622 [~U$ 2,984]).

^c^Responsive caregiving subscore correspond to one indicator and was not included in the overall IMAPI.

**Table 2:** Comparative analysis of the prioritization of nurturing care indicators of IMAPI‐D and IMAPI‐M

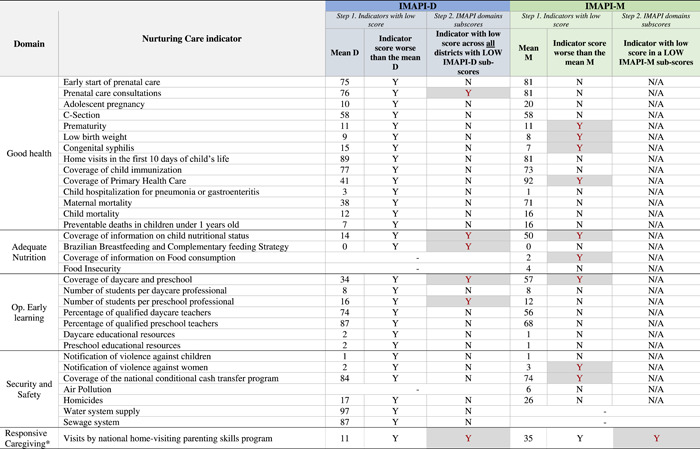

*Note*: Grey shades indicate nurturing care indicators prioritized.

Abbreviations: IMAPI, Brazilian Early Childhood Friendly Municipal Index; IMAPI‐D, IMAPI at the district level; IMAPI‐M, IMAPI at municipal level.

^a^Responsive caregiving subscore correspond to one indicator and was not included in the overall IMAPI.

#### 3.2.2 IMAPI‐D

**Figure 5:**
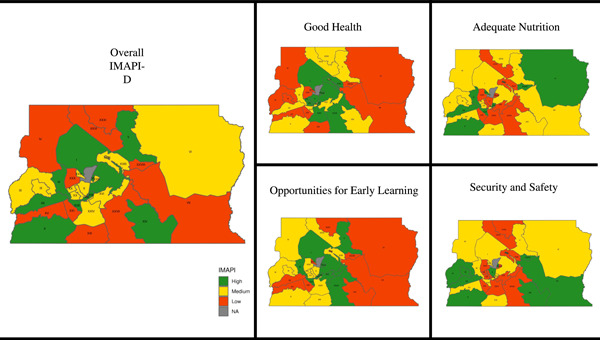
Spatial distribution of overall IMAPI and the four domains subscores in the districts of Brasilia. IMAPI, Brazilian Early Childhood Friendly Municipal Index

The overall IMAPI‐D score and domain subscores across districts are presented in Figure 5 (maps). *Cangolândia* had the highest overall IMAPI‐D and *Fercal* had the lowest. Only one district (*Riacho Fundo II*) had low IMAPI subscores for the four domains of nurturing care, and one district (*Vicente Pires*) had low IMAPI in three domains (Table 1). Six low scoring nurturing care indicators were prioritized: one in the Good health domain (pre‐natal care consultations), two in the Adequate nutrition (coverage of information on child nutritional status as well as the Brazilian Breastfeeding and complementary feeding strategy), two in Opportunities for early learning (coverage of daycare and pre‐school as well as the number of students per pre‐school professional), and one in Responsive caregiving. No indicators were prioritized in the Security and Safety domain (Table 2).

#### 3.2.3 Analysis of prioritized indicators

In total, 14 nurturing care indicators were prioritized across IMAPI‐M (*n* = 12) and IMAPI‐D (*n* = 6) analyses (Table 3). Among those, five indicators were from the Good health domain, three from the Adequate nutrition domain, three from Opportunities for early learning domain, two from the Security and Safety domain, and one from the Responsive caregiving domain. For the socioecological model classification, three of the nurturing care indicators were related to Support Services. Interestingly, Families and Caregivers’ capabilities together were also represented by three indicators. Regarding the role of indicators in the nurturing care environment, four were related to Quality or Coverage, followed by Results (*n* = 3) and Efforts (*n* = 1). Most of the nurturing care indicators prioritized by the IMAPI‐M analysis were related to Coverage (*n* = 5 out of 12), while the IMAPI‐D analysis prioritized two indicators of each Effort, Coverage, and Quality.

**Table 3:** Prioritized nurturing care indicators in IMAPI‐D and IMAPI‐M classified following the analytical model

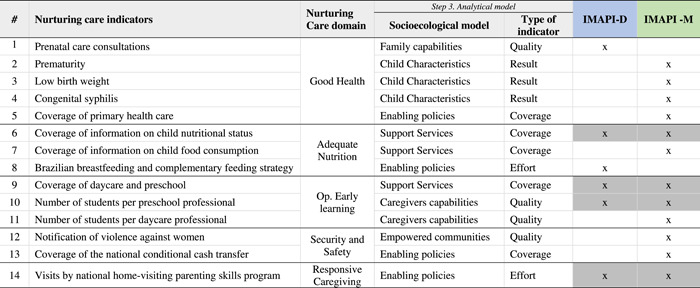

*Note*: Grey shade indicates that indicators prioritized for both IMAPI analyses.

Abbreviations: IMAPI, Brazilian Early Childhood Friendly Municipal Index; IMAPI‐D, IMAPI at the district level; IMAPI‐M, IMAPI at municipal level.

Only four nurturing care indicators were identified as a priority in both IMAPI analyses at the municipal and district levels: one from Adequate nutrition, two from the Opportunities for early learning domains, and one from Responsive caregiving. These four indicators were mapped as enabling policies, supportive services, and caregivers’ capabilities (socioecological model) and Effort, Coverage, and Quality (indicator's role) (Table 3).

## 4 DISCUSSION

We documented for the first time the strengths and weaknesses of the enabling nurturing care environment at the municipal and district level using the IMAPI approach, grounded in the NCF. We found that using IMAPI at different levels of nurturing care data disaggregation, municipal versus district in this instance can jointly strengthen the ability of decision‐makers to identify nurturing care indicators with low scores that require attention. Moreover, the quantitative prioritization process of nurturing care indicators with low scores and the innovative analytical framework combining the allocation of the indicators into the socioecological model and the role of indicators in the enabling nurturing care environment proved to be a sound process to identify gaps that will require investments to improve ECD outcomes among the most vulnerable.

The two analyses of IMAPI conducted in this study, at municipal and district disaggregation levels, together prioritized 14 low scoring nurturing care indicators, with only 4 common nurturing indicators being prioritized. Despite the low overlap across IMAPI analyzes on the prioritized nurturing care indicators, the framework helped to identify the nature of the gaps to strengthen the nurturing care environment for ECD (Nurturing Care Framework for Early Childhood Development, 2021b). Across socioecological model levels, the common prioritized indicators were related to Efforts to enable policies, Coverage of Supportive services, and Quality of Caregivers’ capabilities. Our findings strongly indicate that effective investments in health, nutrition, and education services are needed to enable the nurturing care environment for ECD in Brasilia. Likewise, investments will be needed to enhance caregivers’ qualifications and capabilities to provide early stimulation and opportunities for learning, as these have been described as a way to protect and promote responsive caregiving (Hentschel et al., 2021). This is particularly important in large urban metropolitan areas such as Brasilia, where the majority of families do not have the support of extended families or live far from relatives, most women work outside the home, and must rely on responsive care services within the community (Dai, 2019; Hays, 1996; Moussié, 2021).

Across the role of indicators in the nurturing care environment, five indicators were Coverage, four Quality, three Results, and two Efforts. Investments in enabling the implementation and expanding the Coverage of ECD nurturing care policies and programmes have been a strategy largely used in low‐ and middle‐income countries (Kofke et al., 2021; Pérez‐Escamilla et al., 2018). In contrast, most of them have rarely invested in quality control and monitoring systems and in long‐term results (Kofke et al., 2021; Pérez‐Escamilla et al., 2018). Specifically, in Brazil, the *Programa Crianca Feliz* (PCF) is an example of a nurturing care programme that has received major investments to become a large‐scale programme within a short timeframe; however, PCF has not had strong monitoring and accountability of its Quality, which could be hampering effective results (Buccini, Venancio, et al., 2021). Hence, our findings confirm the critical role of investing in the Quality and result of scaling up nurturing care services to enhance early learning and responsive caregiving.

The IMAPI‐M at the municipal level prioritized more indicators of Coverage, which reflects the effort of the municipality to offer ECD‐nurturing care‐related policies, programmes, and services (Albuquerque & Martins, 2017; Pan American Health Organization, 2018). This is consistent with a municipality with a high overall IMAPI‐M score and without low domain subscores, such as Brasilia. In this context, investments in Coverage of services may help to reach the most vulnerable children in the municipality. However, the next step moving forward must include investment in the Quality of services to meet the demand of families for programmes and services with effective results (Albuquerque & Martins, 2017; Pan American Health Organization, 2018), which is consistent with IMAPI‐D that prioritized more indicators of Coverage and Quality. In this context, investments in gaining scale with Quality of programmes and services may help to reduce nurturing care inequities within and across districts (Torres et al., 2018). Hence, the joint information generated through the application of IMAPI analyses allowed a more in‐depth understanding of the enabling environment for ECD in Brasilia than would have been the case if only one level of disaggregation had been conducted.

This study helped us to generate hypotheses to understand why different indicators were prioritized in each of the two analyses, that is, the municipal and district levels. Our first hypothesis is that the causal pathways and interconnectedness of prioritized indicators should be seen within and across the nurturing care domains. Our second hypothesis is that the socioecological model and the role of the indicator in the enabling environment should also be incorporated into the understanding of causal pathways. For example, within the Good health domain, there was no overlap in indicators prioritized by the IMAPI‐M and the IMAPI‐D analyses. However, indicators of Quality, for example, pre‐natal care consultations (prioritized by the IMAPI‐D), can improve indicators of Results, such as prematurity, low birthweight, and congenital syphilis (prioritized by IMAPI‐M) (Albuquerque & Martins, 2017). Furthermore, expanding the coverage of primary health care (prioritized by IMAPI‐M) can facilitate these Supportive services to reach more people and change the indicators of Results (Albuquerque & Martins, 2017). In the Adequate nutrition domain, prioritized low scoring nurturing care indicators indicated that Coverage and Efforts to implement existing policies are needed to strengthen nutrition support services. The low Coverage of the Brazilian Food and Nutrition Surveillance System (SISVAN), which collects continuous information about the nutritional status and food consumption of children and adolescents receiving primary health care services, has been reported previously (Mourão et al., 2020; Nascimento et al., 2017). Consistent with our analysis, higher Coverage of SISVAN has been identified in the most economically vulnerable regions, which may be explained by the need to complete SISVAN as one of the requirements for families to receive the Brazilian conditional cash transfer (Nascimento et al., 2017). Likewise, Brasilia still requires work to implement the Brazilian Breastfeeding and Complementary Feeding Strategy (EAAB), which aims to qualify primary care health professionals on the topics of breastfeeding and complementary feeding, and the PCF, the national home‐visiting parenting skills programme. Confirming our findings, the EAAB has been implemented in only 3.5% of the primary health care centres and the PCF in only 7 out of 30 districts; both programmes have faced challenges to sustain the implementation efforts and reach scale with Quality (Bortolini, 2017; Tavares et al., 2018). Hence, IMAPI analysis in Brasilia evidenced the importance of strengthening nutritional and responsive caregiving services to enable the nurturing care environment for ECD.

The quantitative prioritization process was limited to the application in a single setting; that is, Brasilia. However, the decision flowchart developed from our study may be replicable in any municipality with nurturing care indicators and IMAPI scores. We also acknowledge that moving forward the quantitative prioritization process should be complemented by interviews with multi‐sectoral stakeholders in the municipality and districts. The inclusion of the Responsive caregiving domain can be considered a strength of the quantitative prioritization process. Originally, the Responsive caregiving domain was not included in the IMAPI due to the statistical limitation of calculating a subscore with a single indicator (Buccini, Pedroso, et al., 2021). Challenges in measuring and prioritizing responsive caregiving have been previously reported (Hentschel et al., 2021). Therefore, incorporating Responsive caregiving in the quantitative prioritization process acknowledges the prioritization and investment in this domain as a foundational component of the NCF without diminishing the need to better measure this domain in future studies. The goal of the analytical framework developed in this study was to support interpretation of IMAPI findings and increase the understanding of decision‐makers about the causal pathways and interconnectedness within and across the nurturing care domains. Therefore, the gaps in the combination of socioecological levels and the role of indicators can guide the definition of new process indicators to improve the measurement of the enabling environment for nurturing care.

Due to the effort and investment to build the IMAPI at the district level, trade‐offs between municipal and district analysis should be considered. First, it is important to consider whether districts would have the autonomy to implement and fund a tailored action and accountability plan. Second, to conduct a comparative analysis as we did in this manuscript, it is important to consider data available at both the municipality and district levels. One limitation in the comparison between IMAPI‐M and IMAPI‐D was the different number of indicators in the Adequate nutrition domain and in the Security and Safety domain. Specifically, the Security and Safety domain was the one that had the most differences when comparing both analyses. As a result, indicators in this domain were prioritized only by the IMAPI‐M analysis. Therefore, before collecting IMAPI data at the district level, we recommend (i) defining the purpose of the analysis, whether it is to pursue a comparative analysis (municipal vs. district) or to use both to select common prioritized indicators as presented in this manuscript, and (ii) discussing with managers and investors the pros and cons of including or removing indicators that may be or may not be available at the district level, as this can impact the prioritization process, as noted in the current analysis for the Security and Safety domain. Despite considerations and potential limitations, we found that in the case of urban territorial inequalities, disaggregated nurturing care data at different levels can inform a meaningful prioritization process.

In summary, combining findings from different levels of IMAPI analyses, municipal and district levels, provides a powerful tool to prioritize investments and monitor the nurturing care environment within metropolitan areas. This is particularly true when district levels within large metropolitan areas have the autonomy to make their own investment decisions. Future qualitative research with decision‐makers is needed to further understand the utility of disaggregating IMAPI scores at different levels of political administration.

## CONFLICT OF INTERESTS

The authors declare that there are no conflict of interests.

## AUTHOR CONTRIBUTIONS

GB and MBG conceptualized the prioritization process of nurturing care indicators at different levels of government. GB analysed the data with the support of JP and JLP. SEACK and JP coordinated the efforts of data collection of indicators in Brasilia. MEB, AS, JB computed IMAPI scores. GB interpreted the data, developed the first draft of the manuscript with intellectual inputs from MBG and RPE both, revised it critically through an iterative process. All authors revised and approved the final version of the manuscript.

## Supporting information

Supplementary information.

Supplementary information.

## Data Availability

The data that support the findings of this study are available from the corresponding author upon reasonable request.
