# Supplementary material for: Prioritizing nurturing care at the municipal and district level with the Brazilian Early Childhood Friendly Municipal Index (IMAPI)
Source: Matern Child Nutr. 2022 Mar 7;18(Suppl 2):e13312. doi: 10.1111/mcn.13312 (PMC8968938; doi:10.1111/mcn.13312)
Supplement: Supplementary file 1 — Supplementary information. [file MCN-18-e13312-s001.docx]

Appendix 1. Definition of IMAPI's nurturing Care indicators

|  | | |  | |
| --- | --- | --- | --- | --- |
| **Domain** | **Nurturing Care indicator** | **IMAPI-D definition** | **IMAPI-M definition** |  |
| Good health | Early start of prenatal care | Total pregnant women who started prenatal care in the first 3 months or less of pregnancy in relation to the total number of pregnant women monitored. by year and district of residence. | Total pregnant women who started prenatal care in the first12 weeks of pregnancy in relation to the total number of pregnant women monitored. by year and municipality of residence. |  |
|  | Prenatal care consultations | Number of pregnant women with 7 or more prenatal care consultations in relation to the total number of pregnant women monitored (number of live births). by year and district of residence. | Number of pregnant women with six or more prenatal care consultations in relation to the total number of pregnant women monitored (number of live births). by year and municipality of residence. |  |
|  | Adolescent pregnancy | Prevalence of pregnant adolescents (10 to 19 years). by year and district of residence. | Prevalence of pregnant adolescents (10–19 years). by year and municipality of residence. |  |
|  | C-Section | Number of births via C-Section in relation to the total number of births. by year and district of residence. | Number of births via C-section in relation to the total number of births. by year and municipality of residence. |  |
|  | Prematurity | Percentage of children born at less than 37 completed weeks of gestation in relation to the total number of births. by year and district of residence. | Percentage of children born at less than 37 completed weeks of gestation in relation to the total number of births. by year and municipality of residence. |  |
|  | Low birth weight | Percentage of live births weighing less than 2500 g in relation to total births. by year and district of residence. | Percentage of live births weighing less than 2500 g in relation to the total number of births. by year and municipality of residence. |  |
|  | Congenital syphilis | Number of confirmed and notified cases of congenital syphilis in children under 5 years old in relation to the total number of children under 5 years old. by year and district of residence. | Number of confirmed and notified cases of congenital syphilis in children under 5 years old in relation to the total number of children under 5 years old. by year and municipality of residence. |  |
|  | Home visits in the first 10 days of child’s life | Percentage of primary care teams in the district that participated in the second cycle of the PMAQ (National Program for Improving Access and Quality in Primary Care) and reported carrying out home visits in the first 10 days of child’s life. | Percentage of primary care teams in the municipality that participated in the second cycle of the PMAQ (national programme for improving access and quality in primary care) and reported carrying out home visits in thefirst 10 days of child'slife. |  |
|  | Coverage of child immunization | Percentage of children who received the tetra viral vaccine. by year and district. | Percentage of children who received first dose of DTaP immunization (triplebacterial vaccine). by year and municipality of residence. |  |
|  | Coverage of Primary Health Care | Percentage of the population covered by Primary Health Care. by year and district. | Percentage of the population covered by primary health care. by year and municipality. |  |
|  | Child hospitalization for pneumonia or gastroenteritis | Percentage of hospitalization of children under 5 years with pneumonia or gastroenteritis in relation to the total number of children under 5 years. by year and district of residence. | Percentage of children under 5 years old  hospitalized for pneumonia or gastroenteritis in relation to the total number of children under 5 years old. by year and municipality of residence. |  |
|  | Maternal mortality | Number of women who died from causes related to pregnancy for every 100.000 live births. per year and district of residence. | Number of women who died from causes related to pregnancy. childbirth or puerperium for every 100.000 live births. per year and municipality of residence. |  |
|  | Child mortality | Number of deaths of children under 5 years old for every 1000 live births. per year and district of residence | Number of deaths of children under 5 year sold for every 1000 livebirths. per year andmunicipality ofresidence. |  |
|  | Preventable deaths in children under 1 years old | Deaths that could be prevented by the performance of health services in children under one year old in relation to the total number of live births. by year and district of residence. Deaths caused by the following categories are considered “preventable”: a) Reducible by immunizations; b) Reducible by caring for women during pregnancy; c) Reducible by adequate care for women during childbirth; d) Reducible by actions. diagnosis. and appropriate treatment; e) Reducible by health promotion activities linked to Primary Health Care. | Deaths that could be prevented by the performance of healthcare services in children under 1 year old in relation to the total number of live births. by year and municipality of residence. Deaths caused by the following categories are considered ‘preventable’:(a) reducible by immunizations;(b) reducible by caring for women during pregnancy; (c) reducible by adequate care for women during childbirth; (d) reducible by actions. diagnosis and appropriate treatment; and  (e) reducible by health promotion activities linked to primary healthcare. |  |
| Adequate nutrition | Coverage of information on child nutritional status | Percentage of children under 5 years of age with at least one record of information on nutritional status (BMI/age) in the Food and Nutrition Surveillance System (SISVAN). by year and district of residence. | Percentage of children under 5 years of age with at least one record of information on nutritional status (BMI/age) in the food and nutrition surveillance system (SISVAN). by year and municipality of residence. |  |
|  | Brazilian Breastfeeding and Feeding Strategy | The indicator Brazilian Breastfeeding and Feeding Strategy (EAAB) is an indicator composed a workshop and/or training for instructors and/or certification of the basic health unit in the district. | The indicator Brazilian breastfeeding and  feeding strategy (EAAB) is an indicator  composed a workshop and/or training for  instructors and/or certification of the basic  health unit in the municipality. |  |
|  | Coverage of information on child food consumption | - | Percentage of children under 5 years old with at least one record of information on food consumption (breastfeeding. quality. and diversity of the diet) in the Food and Nutrition Surveillance System (SISVAN). by year and municipality of residence. |  |
|  | Severe food insecurity | - | Estimated prevalence of severe food insecurity in a given municipality. Severe food insecurity is characterized by children’s quantitative reduction in food. disruption in eating patterns resulting from lack of food. and hunger caused by the inability to buy food due to the lack of money. |  |
| Op. Early learning | Coverage of daycare and preschool | Number of enrollments in public and private daycares and preschools in relation to the total number of children under 5 years. by year and district. | Number of enrolments in daycare and preschool in relation to the total number of children under 5 years. by year and municipality of residence. |  |
|  | Number of students per daycare professional | Number of students enrolled for each professional employed in public and private daycares in the district per year. | Number of students enrolled for each professional employed in public and private daycares in the municipality per year. |  |
|  | Number of students per preschool professional | Number of students enrolled for each professional employed in public and private preschools in the district per year. | Number of students enrolled for each  professional employed in public and private preschools in the municipality per year. |  |
|  | Percentage of qualified daycare teachers | Percentage of teachers with higher education employed in public and private daycares in the district per year. | Percentage of teachers with higher education employed in public and private daycares in the municipality per year. |  |
|  | Percentage of qualified preschool teachers | Percentage of teachers with higher education employed in public and private preschools in the district per year. | Percentage of teachers with higher education employed in public and private preschools in the municipality per year. |  |
|  | Daycare educational resources | Presence of library/study room and/or playground and/or children’s restroom in public and private daycares in the district per year. | Presence of library/study room and/or playground and/or children's restroom in daycares in the municipality per year. |  |
|  | Preschool educational resources | Presence of library/study room and/or playground and/or children’s restroom public and private preschools in the district per year. | Presence of library/study room and/or  playground and/or children's restroom in  schools for early childhood in the municipality per year. |  |
| Security and Safety | Notification of violence against children | Total reported cases of any type of violence against children under 5 years old in relation to the total number of children under 5 in the district per year. | Total reported cases of any type of violence against children under 5 years old in relation to the total number of children under five in the municipality per year. |  |
|  | Notification of violence against women | Total reported cases of any type of violence against women of childbearing age (10 to 49 years) in relation to the number of women in this age group in the district per year. | Total reported cases of any type of violence against women of child bearing age (10–49 years) in relation to the number of women in this age group in the municipality per year. |  |
|  | Coverage of the national conditional cash transfer program | Percentage of families benefiting from the National conditional cash transfer program among families in the Brazilian single registry with children under 5 years old | Percentage of families benefitting from the national conditional cash transfer programme among families in the Brazilian single registry with children under 5 years old. |  |
|  | Air Pollution | - | Estimated daily concentration of fine particulate matter (PM2.5) (ug/m3) by municipality. |  |
|  | Homicides | Homicide rate estimated per 100 thousand inhabitants for each district. | Homicide rate estimated per 100.000 inhabitants for each municipality. |  |
|  | Water system supply | Coverage of adequate water supply network in the district per year | - |  |
|  | Sewage system | Coverage of adequate sewage system network in the district per year | - |  |
