# Supplementary material for: Prioritizing nurturing care at the municipal and district level with the Brazilian Early Childhood Friendly Municipal Index (IMAPI)
Source: Matern Child Nutr. 2022 Mar 7;18(Suppl 2):e13312. doi: 10.1111/mcn.13312 (PMC8968938; doi:10.1111/mcn.13312)
Supplement: Supplementary file 2 — Supplementary information. [file MCN-18-e13312-s002.docx]

Appendix 2. Identification of indicators with low IMAPI-D scores across Brasilia’s districts organized by Nurturing Care domains. *Notes*: Green indicates scores equal or better than the mean D. Red indicates scores worse than the mean D. Yellow indicates the Districts with Low sub-scores in the domain.

| **Good Health domain** | | | | | | | | | | | | | | |
| --- | --- | --- | --- | --- | --- | --- | --- | --- | --- | --- | --- | --- | --- | --- |
| **Districts** | *Nurturing Care Indicators* | | | | | | | | | | | | | |
|  | **Early start of prenatal care** | **Prenatal care consultations** | **Adolescent pregnancy** | **C-Section** | **Prematurity** | **Low birth weight** | **Congenital syphilis** | **Home visits in the first 10 days of child’s life** | **Coverage of child immunization** | **Coverage of Primary Health Care** | **Child hospitalization for pneumonia or gastroenteritis** | **Maternal mortality** | **Child mortality** | **Preventable deaths in children under 1 years old** |
| Brazlândia | 61.8 | 58.8 | 15.5 | 44.4 | 11.1 | 8.2 | 16.3 | 89.5 | 82.3 | 41.9 | 4.8 | 181.2 | 8.2 | 6.3 |
| SCIA-Estrutural | 68.3 | 65.1 | 25.1 | 38.8 | 10.1 | 10.6 | 45.9 | 88.9 | 98.3 | 72.1 | 2.7 | 0.0 | 23.8 | 12.5 |
| Recanto das Emas | 72.2 | 66.1 | 14.3 | 43.9 | 10.8 | 10.0 | 28.0 | 83.3 | 74.2 | 73.3 | 2.6 | 146.7 | 9.8 | 7.8 |
| Santa Maria | 73.1 | 66.9 | 13.0 | 51.9 | 12.7 | 11.1 | 19.6 | 100.0 | 68.0 | 30.9 | 3.3 | 46.8 | 13.6 | 5.6 |
| Ceilândia | 70.9 | 71.3 | 13.4 | 44.9 | 12.5 | 10.9 | 19.2 | 90.0 | 89.8 | 40.4 | 2.1 | 62.7 | 15.1 | 9.6 |
| Paranoá | 68.0 | 71.6 | 15.3 | 51.3 | 13.7 | 10.4 | 5.2 | 100.0 | 72.7 | 93.8 | 4.1 | 164.2 | 16.4 | 11.5 |
| Planaltina | 73.2 | 71.8 | 15.2 | 42.2 | 9.9 | 7.9 | 47.3 | 64.3 | 71.0 | 54.8 | 4.0 | 0.0 | 12.1 | 4.3 |
| Itapoã | 66.7 | 71.9 | 18.7 | 47.6 | 9.0 | 8.7 | 19.1 | 71.4 | 66.0 | 82.7 | 0.4 | 105.6 | 16.9 | 12.7 |
| Riacho Fundo II | 71.0 | 72.3 | 12.4 | 61.0 | 13.6 | 13.0 | 12.9 | 100.0 | 91.3 | 8.3 | 1.0 | 0.0 | 15.9 | 9.8 |
| Vicente Pires | 77.0 | 72.8 | 6.7 | 69.1 | 13.4 | 10.9 | 16.8 | 89.5 | 51.3 | 25.3 | 0.6 | 0.0 | 10.4 | 5.8 |
| São Sebastião | 71.8 | 73.7 | 12.9 | 49.7 | 11.6 | 9.4 | 10.8 | 89.5 | 49.6 | 79.7 | 1.8 | 0.0 | 10.1 | 6.1 |
| Gama | 81.2 | 73.7 | 11.1 | 54.4 | 12.7 | 11.0 | 20.7 | 95.2 | 99.4 | 48.6 | 4.9 | 48.3 | 15.4 | 8.2 |
| Samambaia | 75.8 | 73.9 | 11.4 | 49.6 | 11.2 | 10.1 | 16.5 | 72.7 | 75.9 | 76.2 | 2.2 | 76.0 | 8.9 | 4.3 |
| Lago Sul | 80.1 | 75.0 | 2.3 | 72.4 | 12.8 | 9.7 | 0.0 | 89.5 | 100.0 | 0.0 | 1.2 | 0.0 | 5.7 | 5.7 |
| Taguatinga | 80.5 | 76.5 | 6.7 | 62.8 | 11.9 | 9.4 | 27.2 | 89.5 | 100.0 | 46.1 | 4.7 | 62.9 | 9.1 | 4.7 |
| NúcleoBandeirante | 76.5 | 77.4 | 9.7 | 60.0 | 11.2 | 9.7 | 7.3 | 89.5 | 78.4 | 24.0 | 2.6 | 0.0 | 12.1 | 7.3 |
| Riacho Fundo | 73.4 | 77.6 | 9.9 | 58.9 | 11.6 | 9.1 | 26.2 | 100.0 | 91.3 | 100.0 | 3.8 | 0.0 | 13.7 | 7.5 |
| Fercal | 76.4 | 77.8 | 18.7 | 40.9 | 14.3 | 9.9 | 11.1 | 66.7 | 73.5 | 100.0 | 0.0 | 0.0 | 14.8 | 4.9 |
| Varjão | 73.0 | 78.8 | 22.8 | 47.1 | 10.6 | 9.5 | 26.2 | 89.5 | 100.0 | 0.0 | 0.0 | 0.0 | 26.5 | 21.2 |
| Cruzeiro | 78.6 | 79.3 | 4.4 | 70.0 | 10.7 | 7.4 | 0.0 | 89.5 | 63.5 | 0.0 | 3.8 | 0.0 | 7.0 | 2.3 |
| Park Way | 79.5 | 79.5 | 3.7 | 64.2 | 9.0 | 6.8 | 0.0 | 89.5 | 78.4 | 33.9 | 3.1 | 0.0 | 21.1 | 5.3 |
| Sobradinho | 77.1 | 80.9 | 10.5 | 59.9 | 11.7 | 8.7 | 24.5 | 100.0 | 73.5 | 57.5 | 7.6 | 75.2 | 9.8 | 4.5 |
| Candangolândia | 77.5 | 81.7 | 8.5 | 60.2 | 10.9 | 7.4 | 0.0 | 89.5 | 95.8 | 37.4 | 4.9 | 0.0 | 10.6 | 7.0 |
| ÁguasClaras | 83.3 | 81.7 | 2.1 | 76.8 | 11.4 | 9.7 | 2.0 | 100.0 | 30.6 | 14.6 | 0.2 | 0.0 | 7.9 | 3.7 |
| Lago Norte | 80.7 | 82.3 | 2.6 | 65.5 | 10.2 | 8.4 | 5.7 | 100.0 | 98.0 | 0.0 | 2.2 | 0.0 | 5.2 | 2.6 |
| Guará | 78.2 | 82.4 | 5.0 | 68.6 | 10.7 | 8.5 | 20.9 | 89.5 | 82.5 | 29.9 | 3.3 | 0.0 | 7.4 | 2.6 |
| Plano Piloto | 79.7 | 82.5 | 3.6 | 68.3 | 11.5 | 9.8 | 1.1 | 89.5 | 71.3 | 3.4 | 3.7 | 0.0 | 5.2 | 1.5 |
| Sobradinho II | 80.9 | 82.7 | 13.2 | 56.6 | 12.6 | 9.4 | 22.1 | 100.0 | 73.5 | 45.3 | 0.7 | 0.0 | 13.7 | 8.8 |
| Jardim Botânico | 81.6 | 84.5 | 1.0 | 70.9 | 12.0 | 8.7 | 0.0 | 89.5 | 49.6 | 0.0 | 0.2 | 0.0 | 12.9 | 6.5 |
| Sudoeste-Octogonal | 84.0 | 89.9 | 1.3 | 76.8 | 10.1 | 10.7 | 0.0 | 89.5 | 63.5 | 0.0 | 0.4 | 160.3 | 8.0 | 3.2 |
| Mean | 75.7 | 76.0 | 10.4 | 57.6 | 11.5 | 9.5 | 15.1 | 89.5 | 77.1 | 40.7 | 2.6 | 37.7 | 12.2 | 6.8 |

| **Adequate Nutrition domain** | | |
| --- | --- | --- |
| **Districts** | *Nurturing Care indicators* | |
|  | **Coverage of information on child nutritional status** | **Brazilian Breastfeeding and Feeding Strategy** |
| Cruzeiro | 7.69 | 0.33 |
| Guará | 5.77 | 0.33 |
| Jardim Botânico | - | 0.33 |
| Lago Norte | 4.59 | 0.33 |
| Lago Sul | 0.88 | 0.33 |
| Park Way | 4.33 | 0.33 |
| Riacho Fundo II | 10.71 | 0.33 |
| Sobradinho II | 7.60 | 0.33 |
| Sudoeste-Octogonal | - | 0.33 |
| Vicente Pires | 4.03 | 0.33 |
| ÁguasClaras | 2.18 | 0.67 |
| Ceilândia | 10.69 | 0.67 |
| Gama | 12.06 | 1.00 |
| Plano Piloto | 4.47 | 0.67 |
| Taguatinga | 10.86 | 1.00 |
| Brazlândia | 24.70 | 0.33 |
| Candangolândia | 17.55 | 1.00 |
| Fercal | 25.36 | 0.33 |
| Itapoã | 23.74 | 0.67 |
| NúcleoBandeirante | 20.66 | 0.33 |
| Paranoá | 14.03 | 0.67 |
| Planaltina | 17.98 | 0.67 |
| Recanto das Emas | 13.92 | 0.67 |
| Riacho Fundo | 23.88 | 0.67 |
| Samambaia | 15.04 | 1.00 |
| Santa Maria | 19.95 | 0.33 |
| São Sebastião | 14.35 | 0.67 |
| SCIA-Estrutural | 36.19 | 0.67 |
| Sobradinho | 20.07 | 0.33 |
| Varjão | 43.64 | 0.33 |
| Mean | 13.90 | 0.53 |

| **Opportunities for early learning domain** | | | | | | | |
| --- | --- | --- | --- | --- | --- | --- | --- |
| **Districts** | *Nurturing Care indicators* | | | | | | |
|  | **Coverage of daycare and preschool** | **Number of students per daycare professional** | **Number of students per preschool professional** | **Percentage of qualified daycare teachers** | **Percentage of qualified preschool teachers** | **Daycare educational resources** | **Preschool educational resources** |
| Fercal | 16.4 | - | 18.3 | - | 94.4 | - | 1.6 |
| Itapoã | 1.0 | - | 25.0 | - | 100.0 | - | 2.0 |
| Paranoá | 21.0 | 13.7 | 19.8 | 86.7 | 90.0 | 2.6 | 2.0 |
| Planaltina | 18.7 | 7.3 | 18.9 | 60.3 | 88.9 | 2.6 | 2.4 |
| Recanto das Emas | 20.5 | 8.4 | 17.1 | 67.3 | 86.4 | 2.8 | 2.6 |
| Riacho Fundo II | 8.2 | 9.5 | 20.0 | 75.0 | 93.5 | 2.8 | 2.8 |
| Samambaia | 27.4 | 11.9 | 19.9 | 80.2 | 85.2 | 2.7 | 2.6 |
| São Sebastião | 17.4 | 7.9 | 21.6 | 76.9 | 89.6 | 2.8 | 2.3 |
| SCIA-Estrutural | 14.6 | 10.3 | 21.5 | 100.0 | 82.9 | 2.0 | 2.5 |
| Varjão | 32.4 | 6.5 | - | 100.0 | - | 3.0 | - |
| ÁguasClaras | 12.6 | 11.7 | 13.8 | 87.9 | 88.6 | 2.9 | 2.9 |
| Brazlândia | 42.4 | 8.1 | 16.3 | 77.8 | 81.8 | 2.6 | 2.4 |
| Candangolândia | 42.6 | 7.6 | 17.2 | 93.3 | 95.0 | 2.5 | 2.7 |
| Ceilândia | 22.1 | 7.6 | 18.2 | 76.7 | 84.1 | 2.7 | 2.6 |
| Cruzeiro | 67.1 | 9.2 | 15.0 | 82.9 | 95.5 | 2.4 | 2.6 |
| Gama | 34.0 | 10.4 | 15.7 | 73.0 | 87.6 | 2.7 | 2.7 |
| Guará | 25.2 | 8.2 | 14.1 | 73.6 | 83.2 | 2.6 | 2.7 |
| Jardim Botânico | 1.5 | 12.5 | 7.0 | 75.0 | 100.0 | 3.0 | 3.0 |
| Lago Norte | 44.9 | 9.0 | 20.0 | 74.3 | 87.5 | 2.7 | 2.5 |
| Lago Sul | 100.0 | 6.4 | 11.4 | 75.0 | 87.6 | 2.9 | 2.9 |
| NúcleoBandeirante | 31.8 | 7.3 | 16.9 | 76.9 | 94.1 | 3.0 | 3.0 |
| Park Way | 88.1 | 11.6 | 17.0 | 65.1 | 92.3 | 2.4 | 2.6 |
| Plano Piloto | 92.5 | 7.8 | 12.5 | 78.2 | 87.3 | 2.8 | 2.8 |
| Riacho Fundo | 33.8 | 8.9 | 16.7 | 90.0 | 93.0 | 2.8 | 2.8 |
| Santa Maria | 34.1 | 10.1 | 18.3 | 88.8 | 92.6 | 2.5 | 2.7 |
| Sobradinho | 47.3 | 5.6 | 14.6 | 70.9 | 87.1 | 3.0 | 2.7 |
| Sobradinho II | 15.0 | 7.6 | 16.6 | 80.0 | 89.3 | 2.4 | 2.5 |
| Sudoeste-Octogonal | 15.2 | 5.6 | 8.8 | 84.6 | 87.9 | 3.0 | 3.0 |
| Taguatinga | 47.7 | 6.9 | 14.2 | 84.9 | 88.9 | 2.8 | 2.9 |
| Vicente Pires | 14.1 | 8.7 | 11.6 | 83.8 | 73.6 | 2.9 | 2.9 |
| Mean | 33.6 | 8.2 | 16.1 | 74.3 | 86.7 | 2.5 | 2.5 |

| **Security and Safety** | | | | | | |
| --- | --- | --- | --- | --- | --- | --- |
| **Districts** | *Nurturing Care indicators* | | | | | |
|  | **Notification of violence against children** | **Notification of violence against women** | **Coverage of the national conditional cash transfer program** | **Homicides** | **Water system supply** | **Sewage system** |
| Vicente Pires | 0.2 | 0.7 | 72.7 | 8.0 | 99.2 | 69.1 |
| Sudoeste-Octogonal | 0.3 | 0.1 | 75.0 | - | 100.0 | 100.0 |
| Park Way | 0.9 | 0.4 | 79.4 | - | 99.5 | 59.6 |
| Sobradinho II | 0.2 | 0.5 | 79.9 | 30.0 | 87.3 | 50.2 |
| Lago Sul | 0.9 | 0.1 | 80.0 | 3.0 | 98.4 | 94.8 |
| Riacho Fundo II | 0.2 | 0.9 | 84.4 | 9.0 | 99.9 | 95.4 |
| Fercal | - | 0.7 | 84.5 | 86.0 | 69.3 | 19.4 |
| Lago Norte | - | 0.3 | 86.5 | 3.0 | 92.6 | 85.2 |
| ÁguasClaras | 0.3 | 0.8 | 86.9 | 4.0 | 99.6 | 94.0 |
| Jardim Botânico | - | 0.4 | 100.0 | - | 81.3 | 50.4 |
| Santa Maria | 1.9 | 2.9 | 80.0 | 27.0 | 98.5 | 98.2 |
| Itapoã | 2.5 | 3.3 | 80.6 | 35.0 | 99.6 | 95.9 |
| Recanto das Emas | 1.4 | 2.1 | 82.0 | 15.0 | 99.6 | 95.8 |
| Ceilândia | 3.0 | 2.1 | 82.5 | 18.0 | 99.7 | 90.4 |
| Planaltina | 1.9 | 1.3 | 83.1 | 31.0 | 99.7 | 89.0 |
| Guará | 2.2 | 2.0 | 84.0 | 2.0 | 99.9 | 98.8 |
| Gama | 3.2 | 2.5 | 84.4 | 27.0 | 96.5 | 93.5 |
| Samambaia | 1.7 | 3.1 | 84.4 | 21.0 | 100.0 | 99.7 |
| São Sebastião | 1.6 | 3.6 | 84.6 | 20.0 | 99.8 | 99.0 |
| Taguatinga | 1.5 | 1.7 | 85.0 | 9.0 | 99.9 | 100.0 |
| Paranoá | 2.7 | 3.6 | 85.3 | 15.0 | 99.3 | 99.7 |
| SCIA-Estrutural | 3.5 | 4.3 | 86.0 | 54.0 | 86.9 | 63.0 |
| Cruzeiro | 3.1 | 1.8 | 86.1 | - | 99.6 | 99.7 |
| NúcleoBandeirante | 0.7 | 2.2 | 86.2 | 4.0 | 98.7 | 94.6 |
| Plano Piloto | 1.1 | 1.1 | 86.4 | 8.0 | 99.8 | 99.0 |
| Sobradinho | 2.9 | 2.2 | 86.7 | 18.0 | 94.3 | 83.1 |
| Brazlândia | 1.6 | 0.9 | 87.1 | 17.0 | 99.5 | 99.6 |
| Varjão | 1.3 | 2.8 | 88.5 | 11.0 | 100.0 | 99.8 |
| Candangolândia | 3.0 | 1.6 | 89.2 | 30.0 | 100.0 | 96.7 |
| Riacho Fundo | 1.7 | 2.3 | 89.4 | 12.0 | 100.0 | 92.6 |
| Mean | 1.5 | 1.7 | 84.3 | 17.2 | 96.6 | 86.9 |

| **Responsive caregiving** | |
| --- | --- |
| **Districts** | *Nurturing care indicator* |
|  | **Visits by national home-visiting parenting skills program** |
| Águas Claras | 0 |
| Brazlândia | 0 |
| Candangolândia | 0 |
| Cruzeiro | 0 |
| Fercal | 0 |
| Gama | 0 |
| Guará | 0 |
| Itapoã | 0 |
| Jardim Botânico | 0 |
| Lago Norte | 0 |
| Lago Sul | 0 |
| Núcleo Bandeirante | 0 |
| Paranoá | 0 |
| Park Way | 0 |
| Planaltina | 0 |
| Plano Piloto | 0 |
| São Sebastião | 0 |
| Sobradinho | 0 |
| Sobradinho II | 0 |
| Sudoeste-Octogonal | 0 |
| Varjão | 0 |
| Vicente Pires | 0 |
| Taguatinga | 21.85 |
| Riacho Fundo II | 33.82 |
| Santa Maria | 39.87 |
| SCIA-Estrutural | 41.67 |
| Ceilândia | 42.16 |
| Riacho Fundo | 44.12 |
| Samambaia | 47.06 |
| Recanto das Emas | 61.76 |
| **Mean** | 11.08 |
